# Effects of Temperature and Light on Microalgal Growth and Nutrient Removal in Turtle Aquaculture Wastewater

**DOI:** 10.3390/biology13110901

**Published:** 2024-11-05

**Authors:** Xiaosong Tian, Xiaoai Lin, Qing Xie, Jinping Liu, Longzao Luo

**Affiliations:** 1 Chongqing Vocational Institute of Engineering, College of Resources and Safety, Chongqing 402260, China; terrytian1985@hotmail.com (X.T.); cqxqing@163.com (Q.X.); 2 School of Chemistry and Environmental Science, College of Life Science, Shangrao Normal University, Shangrao 334001, China; 312201@sru.edu.cn (X.L.); 17379706017@163.com (J.L.)

**Keywords:** nutrient removal, *Desmodesmus* sp. CHX1, biomass, temperature, light

## Abstract

The aim of this study was to explore the effects of temperature and light on microalgal growth and nutrient removal in turtle aquaculture wastewater using a single-factor experiment method. Results showed that the growth process of *Desmodesmus* sp. CHX1 in turtle aquaculture wastewater exhibited three stages, namely adaptation, logarithmic, and stable periods. Temperature and light significantly influenced the growth and protein and lipid accumulation of *Desmodesmus* sp. CHX1. The optimal conditions for the growth and biomass accumulation of *Desmodesmus* sp. CHX1 included a temperature of 30 °C, a photoperiod of 24L:0D, and a light intensity of 180 μmol photon/(m^2^·s). Increased temperature, photoperiod, and light intensity enhanced nutrient removal efficiency. Maximum nitrogen removal was achieved at a temperature of 30 °C, a photoperiod of 24L:0D, and a light intensity of 180 μmol photon/(m^2^·s), with the removal efficiency of 86.53%, 97.94%, 99.57%, and 99.15% for ammonia, nitrate, nitrite, and total phosphorus (TP), respectively. Temperature did not significantly affect TP removal, but increased photoperiod and light intensity improved the removal efficiency of TP. The development of microalgae biomass as a feed rich in protein and lipids could address feed shortages and meet the nutritional needs of turtles, offering a feasible solution for large-scale production.

## Simple Summary

The rapid development of turtle breeding leads to the risk of aquaculture wastewater pollution. The microalgal strain *Desmodesmus* sp. CHX1 has shown great potential in removing nutrients from wastewater. This study investigated the effects of temperature and light on the growth of *Desmodesmus* sp. CHX1 and its nutrient removal in turtle aquaculture wastewater to find the optimal conditions for growth and nutrient removal efficiency. This study was relevant to the development of new strategies for the resource utilization of turtle aquaculture wastewater based on microalgae cultivation.

## 1. Introduction

China is a major aquaculture country. During the breeding process, incidents such as arbitrary feeding and non-standard breeding often occur, resulting in a serious excess of nitrogen, phosphorus, and other elements in the breeding water, ultimately leading to high pollution of organic matter concentrations in turtle aquaculture wastewater. High ammonia nitrogen (NH_4_-N) concentrations and chemical oxygen demand in greenhouse turtle aquaculture wastewater can cause serious harm to the surrounding water environment and ecosystem balance [1]. The serious impact of discharged water on the environment has attracted widespread attention, and it is urgent to carry out ecological and harmless treatments. Conventional industrial and ecological treatment methods are frequently employed for treating turtle aquaculture wastewater. These methods can effectively remove suspended solids and nutrients, but there are also problems such as large investment scale, high operating costs, and the failure to reclaim nutrients from wastewater [2]. Hence, it is crucial to pursue an economical and efficient nutrient recycling method from turtle aquaculture wastewater for the sustainable progression of the turtle breeding industry.

Microalgae are a type of phototrophic unicellular organism which has been shown to have high photosynthetic efficiency, fast growth rate, and strong environmental adaptability [3]. Algae growth can directly absorb organic nutrients and inorganic substances in wastewater, promote phosphorus deposition, reduce ammonia nitrogen, and increase the pH value of water [4]. The utilization of microalgae in wastewater treatment has gained recent popularity due to its numerous benefits such as effective nutrient absorption and robust bioremediation capabilities, rendering it suitable for the treatment of diverse wastewater types including municipal, industrial, and agricultural effluents [5]. Moreover, microalgae contribute significantly to CO_2_ sequestration and dissolved oxygen (DO) enhancement in wastewater systems, advancing the pursuit of carbon neutrality. Post-treatment, microalgae retain substantial application potential, such as serving as nutrient-rich feed for animals, biofertilizers, or as biochar for further wastewater treatment and soil amendment purposes. The use of microalgae to treat aquaculture wastewater can not only purify the wastewater and have antibacterial and disinfection effects on the water body but also reduce the pressure on the aquaculture environment and obtain nutritious algal products, providing feed for economic aquatic species and promoting the sustainable development of the aquaculture industry [6].

*Desmodesmus* sp. CHX1 has shown excellent removal ability for nitrogen and phosphorus from piggery wastewater [2,7]. In this study, *Desmodesmus* sp. CHX1 was selected to investigate its potential for nutrient recovery from turtle aquaculture wastewater. The effects of temperature and light conditions on the growth of microalgae, removal of nitrogen and phosphorus, accumulation of lipids and protein, and biomass accumulation were explored, as well was the feasibility of using the microalgae biomass as turtle feed. The results will provide a theoretical basis for nutrient recovery from turtle aquaculture wastewater.

## 2. Materials and Methods

### 2.1. Microalgal Strain and Wastewater

The microalgae used in this experiment was *Desmodesmus* sp. strain CHX1, which was isolated from piggery wastewater [2,8,9]. The turtle aquaculture wastewater was obtained from a turtle farm in Shangrao City, Jiangxi Province, China. The properties of the turtle aquaculture wastewater were as follows: NH_4_-N of 0.71 mg/L, nitrate nitrogen (NO_3_-N) of 35.14 mg/L, nitrite nitrogen (NO_2_-N) of 35.49 mg/L, total phosphorus (TP) of 10.41 mg/L, and pH of 9.12.

### 2.2. Experiment Design

#### 2.2.1. The Effect of Cultivation Time on the Growth of Microalgae

Microalgae cells (0.2 g/L, dry weight) were inoculated into 200 mL of turtle aquaculture wastewater in a 250 mL conical flask, which was then placed in an oscillating light incubator (ZQZY-70CS, Jtliangyou, Changzhou City, China). The light source was fluorescent tubes (T5 Square style, Dibilun, Shenzhen City, China). The temperature, photoperiod, and light intensity were set at 25 °C, 24 h/d, and 180 μmol photon/(m^2^·s), respectively. Biomass of microalgae was determined every day.

#### 2.2.2. Single-Factor Experiment

Microalgae cells (0.2 g/L, dry weight) were inoculated into 200 mL of turtle aquaculture wastewater in a 250 mL conical flask, which was then placed in an oscillating light incubator to conduct single-factor experiments. The light source was fluorescent tubes. Different temperatures (20, 25, and 30 °C), photoperiods (8L:16D, 16L:8D, and 24L:0D), and light intensities (60 μmol photon/(m^2^·s), 120 μmol photon/(m^2^·s), and 180 μmol photon/(m^2^·s)) were set to investigate the effects of temperature and light conditions on the growth, nutrient removal ability, and biomass accumulation of microalgae. The experimentation of each level for the distinct factors was conducted thrice. Microalgae biomass and water quality indicators were measured daily, and chemical composition of microalgae were determined on the 0th and 6th days. The comprehensive experimental conditions are presented in Table 1.

### 2.3. Analytical Method

The concentrations of NO_2_-N, NO_3_-N, NH_4_-N, and TP were determined in adherence to the standard procedures outlined by the American Public Health Association [10]. The determination of biomass was carried out by measuring the optical density (OD) at the wavelength of 690 nm [7]. Protein content was determined in accordance with the method described by Lowry, et al. [11]. Lipid was extracted using the Bligh–Dyer protocoland determined by the gravimetric method [12].

### 2.4. Statistical Analysis

The data were processed and analyzed using Excel 2016 software, and the significant differences between the experimental data of each group were analyzed by one-way ANOVA using IBM SPSS Statistics 25 software, with a significance level of 0.05.

## 3. Results and Discussion

### 3.1. Variations in Microalgal Biomass with Cultivation Time

**Figure 1:**
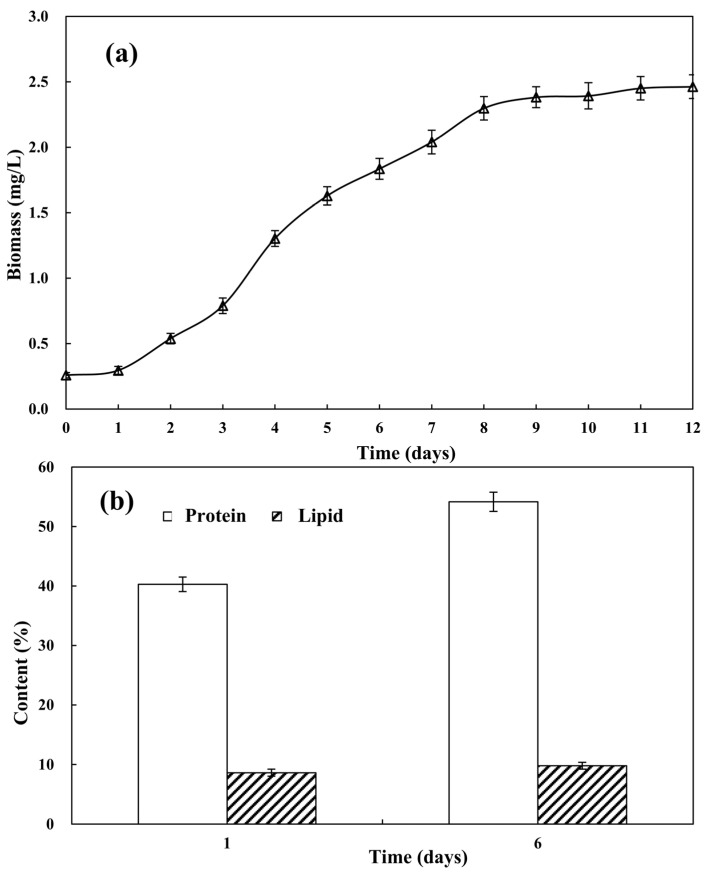
Variations in microalgal biomass (**a**) and protein and lipid accumulation (**b**) with cultivation time (days). Values are means ± standard deviations, *n* = 3.

As shown in Figure 1a, the growth of *Desmodesmus* sp. CHX1 was in the adaptation period from day 1 to day 3. From day 3 to day 9, microalgae cells entered the logarithmic growth period, where they adapted to their growth environment and entered a stage of rapid growth and massive reproduction, with a logarithmic increase in biomass. The growth of *Desmodesmus* sp. CHX1 then entered a stable state from the 9th day. After a period of high-speed growth and reproduction, the microalgae cells consumed more and more nutrients in the wastewater, and harmful substances gradually accumulated [13]. The growth and reproduction of microalgae were limited, and the biomass of the microalgae gradually stabilized. Although these phenomena are consistent with the growth curve of microorganisms, Luo et al. [7] found that there was no obvious adaptation period for the growth of *Desmodesmus* sp. CHX1 in piggery wastewater, and they directly entered the growth period. According to the research of Han, et al. [14], there was no stagnation period in the growth process of *Spirulina* in soy sauce wastewater. This might have been caused by the lower concentrations of NH_4_-N in turtle aquaculture wastewater. Nitrogen is one of the most essential nutrients for the growth process of microorganism, and NH_4_-N is more conducive to direct absorption from wastewater and subsequent use for their own life activities. The lower concentrations of NH_4_-N in turtle aquaculture wastewater provided fewer nutrients for the growth of *Desmodesmus* sp. CHX1 thus requiring the microalgae to adapt for a period of time. After six days of cultivation, the protein and lipid contents of *Desmodesmus* sp. CHX1 showed an increasing trend, rising from the initial 40.28% and 8.63% to 54.16% and 9.80% (Figure 1b), respectively. This indicated that *Desmodesmus* sp. CHX1 cultivated in turtle aquaculture wastewater showed great potential in feed production. After cultivation in wastewater, the protein and lipid contents of *Desmodesmus* sp. CHX1 increased.

### 3.2. Influences of Temperature and Light on the Growth of Microalgae

#### 3.2.1. Growth and Biomass Accumulation of Microalgae at Different Temperatures

**Figure 2:**
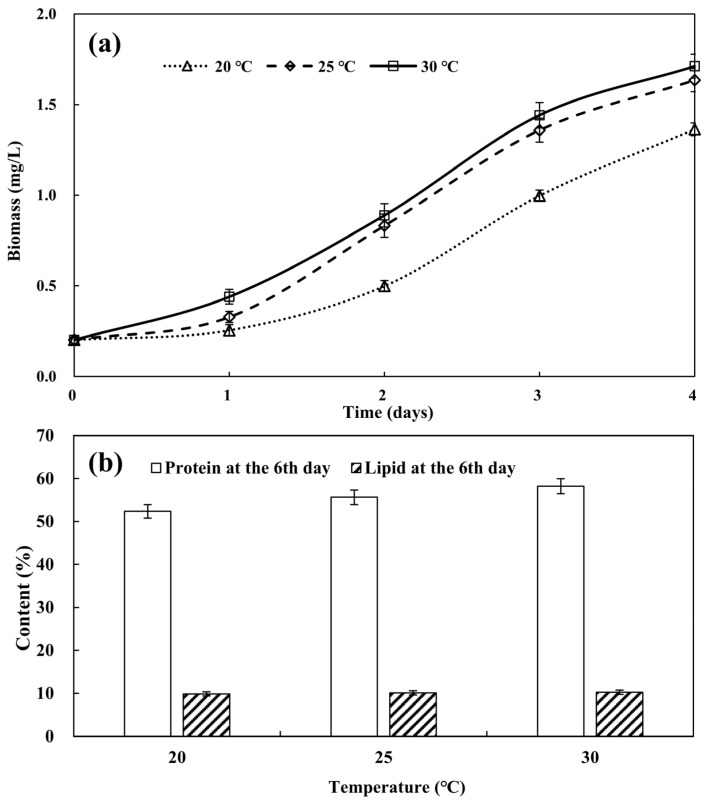
Microalgal biomass (**a**) and protein and lipid accumulation (**b**) at different temperatures. Values are means ± standard deviations, *n* = 3.

Temperature can affect the enzyme activity within microalgae cells, which in turn affects the metabolic processes and growth of microalgae cells [15]. In general, an appropriate temperature helps promote the growth of microalgae, while excessive temperature may cause protein denaturation, leading to enzyme inactivation, metabolic disorders, and even algal cell death [16]. As shown in the research of Converti, et al. [17], the biomass of *Chlorella* decreased by 17% at 35 °C, and significant mortality occurred at 38 °C, while the growth of microalgae was not significantly affected by temperature changes within the temperature range of 25–30 °C. This study investigated the growth of *Desmodesmus* sp. CHX1 under temperatures of 20 °C, 25 °C, and 30 °C. The fastest growth rate of *Desmodesmus* sp. CHX1 was observed at 30 °C (Figure 2a), which was significantly higher than those at 20 °C and 25 °C (*p* < 0.05). Thus, the optimal temperature for the growth of *Desmodesmus* sp. CHX1 in turtle aquaculture wastewater was 30 °C.

The protein content of *Desmodesmus* sp. CHX1 cultivated to the 6th day in turtle aquaculture wastewater at temperatures of 20 °C, 25 °C, and 30 °C were investigated. As shown in Figure 2b, increasing trends of protein content with temperature was observed (*p* < 0.05). This could be attributed to the fact that the microalgae increased the production rate of certain enzymes or proteins to ensure regular metabolic function in the face of high temperature [18]. The highest protein content was found at 30 °C, with a value of 58.20%, which was close to that of *Desmodesmus* sp. CHX1 cultured individually in piggery wastewater [19] but higher than that of *Desmodesmus* sp. CHX1 cocultured with bacteria in piggery wastewater [2]. The lipid content of *Desmodesmus* sp. CHX1 also showed increasing trends with temperature (Figure 2b), albeit the increase was not statistically significant (*p* > 0.05). The accumulation of lipids acts as the crucial reserves, offering energy and structural components for cellular functions and enabling microalgae to withstand high temperature stress [20]. The highest lipid content was found at 30 °C, with a value of 10.24%, which was close to that of *Desmodesmus* sp. CHX1 cultured individually in piggery wastewater [19] but higher than that of *Desmodesmus* sp. CHX1 cocultured with bacteria in piggery wastewater [2].

#### 3.2.2. Growth and Biomass Accumulation of Microalgae at Different Photoperiods

**Figure 3:**
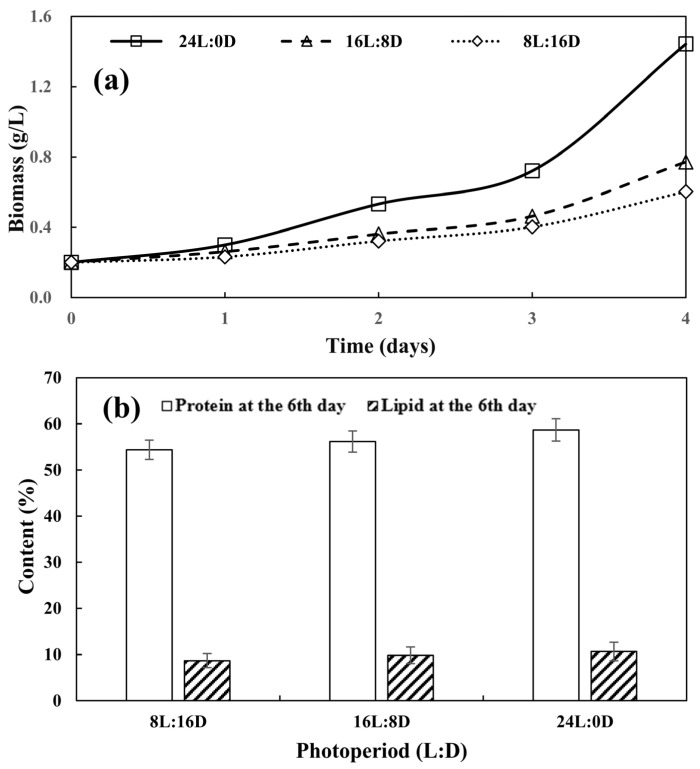
Microalgal biomass (**a**) and protein and lipid accumulation (**b**) at different photoperiods. Values are means ± standard deviations, *n* = 3.

The photoperiod plays a crucial role in determining the growth rate of photoautotrophic microalgae cultures [21]. Figure 3a illustrates the cell growth of *Desmodesmus* sp. CHX1 under different photoperiod conditions. Results showed that the highest biomass concentration was observed under the photoperiod of 24L:0D (*p* < 0.05). This result was consistent with the findings of Wahidin, et al. [22] and Fettah Fettah, Derakhshandeh, Tezcan Un and Mahmoudi [21], who found that the 24:0 photoperiod yielded the greatest growth rate for *Scenedesmus quadricauda*.

Photosynthetic organisms require light to prosper and execute their metabolic functions effectively [23]. In this study, the protein content of *Desmodesmus* sp. CHX1 increased as the light exposure hours were extended (Figure 3b). Peak protein content occurred under a photoperiod of 24L:0D, reaching a protein level of 58.69%, which was significantly higher than those under a photoperiod of 8L:16D and 16L:8D (*p* < 0.05). Bhat, Unpaprom and Ramaraj [23] reported similar variation trends for *Spirulina*, the maximum protein content of which was observed under a photoperiod of 14L:10D. This indicated that increasing the photoperiod and maintaining ideal light intensity for the cultivation process led to an increase in protein content of the microalgae [23]. Figure 3b illustrates the cumulative lipid content in microalgae cultivated under photoperiods of 8L:16D, 16L:8D, and 24L:0D. The highest lipid content (10.65%) was achieved with a photoperiod of 24L:0D after 6 days of cultivation (*p* < 0.05). In comparison, the lipid content was lower (9.83%) under a photoperiod of 18L:6D. A shortened photoperiod of 8L:16D resulted in a reduction in lipid content down to 8.67%. Wahidin, Idris and Shaleh [22] found that the highest lipid content was obtained under a photoperiod of 18L:6D for *Nannochloropsis* sp. Microalgae subjected to different photoperiods undergo significant alterations in their gross chemical constituents, pigment levels, and photosynthetic activity. Moreover, variations in light intensity, along with light and dark rhythm, have been noted to modulate lipid metabolism in microalgae thus varying the lipid composition [24].

#### 3.2.3. Growth and Biomass Accumulation of Microalgae at Different Light Intensities

**Figure 4:**
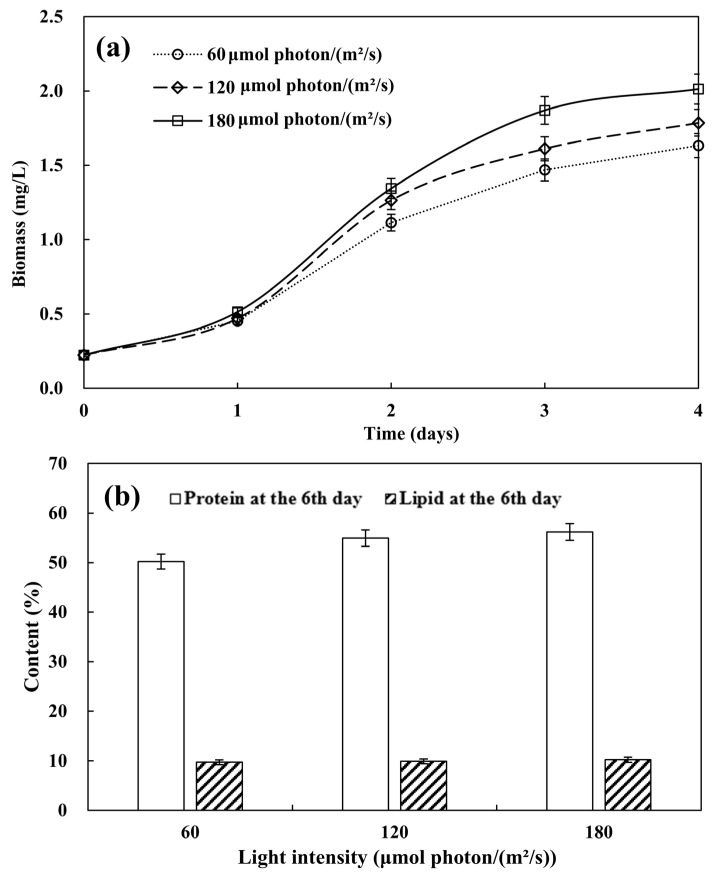
Microalgal biomass (**a**) and protein and lipid accumulation (**b**) at different light intensities. Values are means ± standard deviations, *n* = 3.

Light is an important energy source for microalgae as photosynthetic microorganisms to carry out photosynthesis, produce ATP and NADPH, and synthesize the required molecules. Different light intensities directly affect the photosynthetic activity and growth rate of microalgae. The optimal light intensity for growth and biomass production mainly varies among different algal species. Microalgae usually increase their biomass with the increase in light intensity during the cultivation process. However, under high light conditions, when the saturation point is exceeded, photoinhibition may occur due to the occurrence of intracellular photooxidation reactions [25]. The lighting conditions mainly include the intensity, period, and wavelength of the light. According to the research of Fan et al. [26], microalgae grew faster under strong light conditions, and the consumption of acid soluble polyphosphates also accelerated with increasing light intensity. As shown in Figure 4a, the biomass of microalgae increases with the increasing light intensity (*p* < 0.05). The impact of light intensity on microalgae growth becomes increasingly apparent from the second day. The biomass is highest at a light intensity of 180 μmol photon/(m^2^·s), and lowest at a light intensity of 60 μmol photon/(m^2^·s). An increase in lighting boosts photosynthesis rate at low light intensity, but at saturating intensities, further increases do not raise the rate. Excessive light can cause photo-oxidative damage, leading to photoinhibition in microalgae [27]. The results of this study indicate that the light intensity in this study has not yet reached the saturation point of light intensity for *Desmodesmus* sp. CHX1.

The protein contents of *Desmodesmus* sp. CHX1 cultivated in turtle aquaculture wastewater at light intensities of 60 μmol photon/(m^2^·s), 120 μmol photon/(m^2^·s), and 180 μmol photon/(m^2^·s) were investigated. As shown in Figure 4b, increasing trends of protein content with light intensity are observed (*p* < 0.05). The highest protein content is found at an intensity of 180 μmol photon/(m^2^·s), with a value of 56.18%. On the contrary, decrease in protein content with an increase in light intensity was observed for *Desmodesmus* sp. and *S*. *obliquus* in the study by Nzayisenga, et al. [28]. As shown in Figure 4b, increasing trends of lipid content with light intensity are observed, albeit not statistically significant (*p* > 0.05). Similarly, Nzayisenga, Farge, Groll and Sellstedt [28] found that the lipid content of *Dunaliella tertiolecta* showed an increasing trend with light intensity. Under intense light conditions, an increase in lipid content appears as a stress reaction since the triacylglycerol synthesis pathway acts as an electron sink, utilizing the surplus electrons produced in the photosynthetic electron transport chain for consumption [29].

### 3.3. Influences of Temperature and Light on the Removal of Nutrient by Microalgae

#### 3.3.1. Nutrient Removal at Different Temperatures

**Figure 5:**
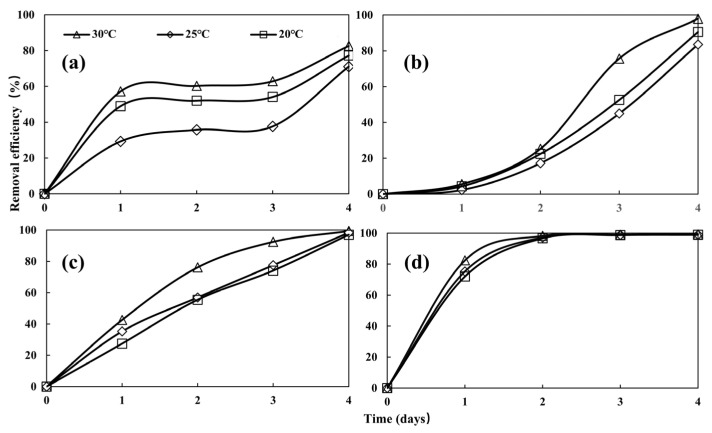
Removal of NH_4_-N (**a**), NO_3_-N (**b**), NO_2_-N (**c**), and TP (**d**) at different temperatures. Values are means ± standard deviations, *n* = 3.

The nitrogen removal efficiencies of *Desmodesmus* sp. CHX1 at temperatures of 20 °C, 25 °C, and 30 °C are shown in Figure 5. Significant differences in the removal efficiency among different temperatures for NH_4_-N and NO_2_-N were observed (*p* < 0.05), while differences in the NO_3_-N removal efficiency among the different temperatures were not significant (*p* > 0.05). The removal efficiency of NH_4_-N, NO_3_-N, and NO_2_-N showed increasing trends with temperature. Similarly, the increased removal of NH_4_-N with higher temperatures was observed for the treatment of municipal wastewater by microalgal–bacteria consortium [30]. The highest removal efficiencies of NH_4_-N, NO_3_-N, and NO_2_-N were observed at 30 °C, with the removal efficiencies of 82.62%, 97.94%, and 99.37%, respectively. The lowest removal efficiencies of NH_4_-N, NO_3_-N, and NO_2_-N were observed at 25 °C, with the removal efficiencies of 77.27%, 83.59%, and 97.02%, respectively. Elevated temperatures boost the metabolic rate in microalgae, thereby improving nutrient removal efficiency when the temperature remains below the ideal level for the growth of microalgae [31]. Therefore, increased temperatures play a significant role in improving nitrogen elimination. In addition, the extent of nitrogen removal is also governed by the growth of algae. Notably, the algae biomass yield was reduced in cooler conditions, leading to diminished nitrogen removal efficiency.

As shown in Figure 5d, the removal efficiencies of TP in each treatment reached the maximum value (>99%) on the second day. There was no significant difference in TP removal efficiency among the treatments (*p* > 0.05), indicating that temperature exerted no significant effect on the removal of TP from turtle aquaculture wastewater by microalgae. This might be due to the low phosphorus concentration in the wastewater, which was easily absorbed by the microalgae [32], resulting in insignificant temperature effects.

#### 3.3.2. Nutrient Removal Under Different Photoperiod Conditions

**Figure 6:**
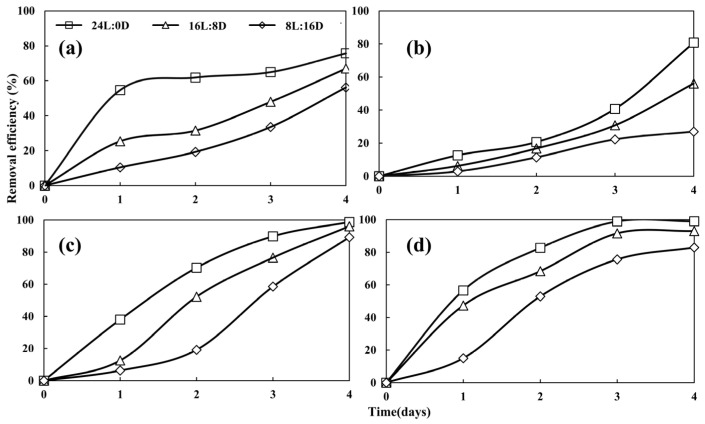
Removal of NH_4_-N (**a**), NO_3_-N (**b**), NO_2_-N (**c**), and TP (**d**) at different photoperiods. Values are means ± standard deviations, *n* = 3.

The nitrogen removal efficiencies of *Desmodesmus* sp. CHX1 under the photoperiods of 8L:16D, 16L:8D, and 24L:0D were significantly different (*p* < 0.05) (Figure 6). The removal efficiency of NH_4_-N, NO_3_-N, and NO_2_-N displayed an upward trend in correlation with the photoperiod. The same variations in NH_4_-N removal efficiency with photoperiod were found in the treatment of municipal wastewater by microalgal–bacteria consortium [33]. Maximum removal rates for NH_4_-N, NO_3_-N, and NO_2_-N were achieved at a photoperiod of 24L:0D, with the respective efficiencies reaching 75.73%, 80.81%, and 98.76%. Conversely, the minimum removal efficiencies for NH_4_-N, NO_3_-N, and NO_2_-N were recorded at a photoperiod of 8L:16D, with efficiencies at 56.20%, 83.59%, and 89.31%, respectively.

The removal of TP under different photoperiods are depicted in Figure 6d. Photoperiod significantly affected the removal of TP by *Desmodesmus* sp. CHX1 (*p* < 0.05). The removal efficiency of TP was positively correlated with the photoperiod; as the photoperiod increased, the corresponding removal efficiency of TP in turtle farming wastewater improved. It is observed that by the fourth day of microalgae cultivation, with a photoperiod of (24L:0D), the removal efficiency of TP reached its peak at 99.01%. In contrast, the removal efficiency of TP for photoperiod of (16L:8D) were somewhat reduced, registering at 93.10% and 82.98%, respectively. Gonçalves, et al. [34] also found that the removal of phosphates increased significantly during extended light exposure, specifically during 24-h light cycles, in contrast to 10-h and 14-h light cycles, while Li, et al. [35] reported that a photoperiod of (12L:12D) was the most effective lighting cycle for treating mariculture wastewater using a bacteria–algae coupled reactor.

#### 3.3.3. Nutrient Removal at Different Light Intensities

**Figure 7:**
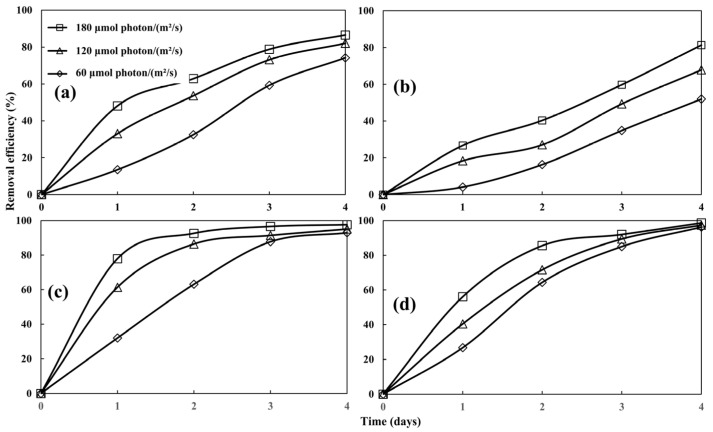
Removal of NH_4_-N (**a**), NO_3_-N (**b**), NO_2_-N (**c**), and TP (**d**) at different light intensities. Values are means ± standard deviations, *n* = 3.

Figure 7 illustrates the nitrogen removal performance of *Desmodesmus* sp. CHX1 at the light intensities of 60 μmol photon/(m^2^·s), 120 μmol photon/(m^2^·s), and 180 μmol photon/(m^2^·s). It is evident that the efficiency in removing NH_4_-N, NO_3_-N, and NO_2_-N increased significantly with the rise in light intensity (*p* < 0.05). This can be attributed to the increase in the biomass of microalgae with the increase in light intensity (Figure 4a). As an increase in lighting boosts photosynthesis rate below the saturation point of light intensity [36], thus more nitrogen is assimilated by microalgae. The maximum removal efficiencies for NH_4_-N, NO_3_-N, and NO_2_-N were achieved at 180 μmol photon/(m^2^·s), reaching 86.53%, 81.37%, and 97.57% respectively, whereas the least removal efficiencies were 74.20% for NH_4_-N, 51.99% for NO_3_-N, and 92.93% for NO_2_-N, at 60 μmol photon/(m^2^·s). Increased light intensity stimulated a rise in the metabolic rate of microalgae, thereby improving nutrient removal for as long as the intensity remained within the optimal threshold for the growth of microalgae. Thus, increased light intensity plays a crucial role in boosting nitrogen removal rates. Additionally, the effectiveness of nitrogen removal is heavily dependent on the rate at which microalgae grow. It is noteworthy that reduced light intensity caused a decline in algal biomass yield, consequently leading to diminished efficiency in nitrogen removal.

The removal of TP under different light intensities are depicted in Figure 7d. The removal efficiency of TP in each treatment showed significant differences in the first two days (*p* < 0.05) as well as increased trends with light intensity. Subsequently, the difference in removal efficiency of TP under different light intensities became smaller and smaller, which was not significant by the 4th day (*p* > 0.05). Variations in TP removal efficiency with light intensity was consistent with the results reported by Nzayisenga, Farge, Groll and Sellstedt [28]. This might have been due to the low phosphorus concentration in the wastewater, which was easily absorbed by the microalgae [32], resulting in insignificant light intensity effects.

### 3.4. Potential of Microalgal Biomass as Feed of Turtle

Based on the current shortage of feed resources, especially protein feed, the development of unconventional feed to promote the reduction in soybean meal and corn has become a research hotspot in recent years. Microalgae, rich in various nutrients such as protein, carbohydrates, fats, minerals, and vitamins, can solve some of the problems of feed resource shortages and have the potential for large-scale production. Taking turtle feed as an example, turtles require a crude protein content of 38–43% and a crude lipid content of about 5% at different growth stages [37]. The protein and lipid contents in the cells of *Desmodesmus* sp. CHX1 cultured in turtle aquaculture wastewater reached around 58% and 10%, which met the nutritional needs of the turtles at different growth stages. This indicates that producing turtle feed by cultivating *Desmodesmus* sp. CHX1 in turtle aquaculture wastewater is feasible.

## 4. Conclusions

This study investigated the effects of temperature and light on the growth and nutrient removal ability of *Desmodesmus* sp. CHX1 in turtle aquaculture wastewater. The growth of *Desmodesmus* sp. CHX1 occurred in the adaptation, logarithmic, and stable periods. Microalgal growth and nutrient removal ability was significantly influenced by temperature and light conditions. Optimal conditions for the growth, protein and lipid accumulation, and nutrient removal were a temperature of 30 °C, a photoperiod of 24L:0D, and a light intensity of 180 μmol photon/(m^2^·s). The removal efficiencies for NH_4_-N, NO_3_-N, NO_2_-N, and TP achieved 86.53%, 97.94%, 99.57%, and 99.15% under the optimal conditions. Cultivating microalgae under these conditions could provide a protein- and lipid-rich feed for turtles, addressing feed shortages in commercial turtle aquaculture.

## Figures and Tables

**Table 1:** Detailed conditions for the single-factor experiments.

Factor	Number	Temperature (°C)	Photoperiod (L:D)	Light Intensity (μmol Photon/(m^2^·s))
Temperature	A1	20	24:0	180
	A2	25	24:0	180
	A3	30	24:0	180
Photoperiod	B1	30	8:16	180
	B2	30	16:8	180
	B3	30	24:0	180
Light intensity	C1	30	24:0	60
	C2	30	24:0	120
	C3	30	24:0	180

## Data Availability

Data will be made available on request.
